# Supplementary material for: Kinetically driven successive sodic and potassic alteration of feldspar
Source: Nat Commun. 2021 Jul 21;12:4435. doi: 10.1038/s41467-021-24628-1 (PMC8295371; doi:10.1038/s41467-021-24628-1)
Supplement: Supplementary file 1 — Supplementary Information [file 41467_2021_24628_MOESM1_ESM.pdf]

Supplementary Information

Kinetically driven successive sodic and potassic alteration of feldspar

Gan Duan[1*], Rahul Ram[1], Yanlu Xing[1, 2], Barbara Etschmann[1] and Joël Brugger[1*]

***Corresponding author. Email: gan.duan@monash.edu; joel.brugger@monash.edu**

This Supplementary Information entry contains:

- Supplementary Methods
  - Scanning Electron Microscopy
  - Electron Microprobe
  - NanoSIMS analysis
  - Raman Spectroscopic Measurements
  - Mineral liberation analyser
- Supplementary Note: Thermodynamic modelling and GEMs Selektor Calculations
- Supplementary Table 1 - Conditions of each experiments
- Supplementary Table 2 - Starting composition of each system used in GEMs modelling
- Supplementary Figure 1 - BSE images of Biotite, fluorite and ilmenite
- Supplementary Figure 2 - Chemical compositions of three different feldspar phases
- Supplementary Figure 3 - MLA results of different feldspar fractions
- Supplementary Figure 4 - ^{18}O and ^{16}O counts in feldspars
- Supplementary Figure 5 - Main Raman spectrum peaks of each phase
- Supplementary Figure 6 - Raman Mappings of the position of Raman-active bending mode at 515 cm^{-1} in feldspars
- Supplementary References

Supplementary Methods

Scanning Electron Microscopy (SEM)

A high-resolution FEI Quanta 3D field emission gun (FEG) scanning electron microscope was used to study the microstructure of the reaction products. The working distance was 10 mm for analysis of the polished epoxy block. The accelerating voltage and beam current used were 15-20 kV and 16 nA. Elemental compositions were determined by energy-dispersive X-ray spectroscopy (EDS). The calculated chemical formula of sanidine from EDS is $K_{0.6}Na_{0.3}Al_{1.1}Si_3O_8$.

Electron Microprobe Analysis (EMPA)

A JEOL JXA-8530F electron microprobe incorporating wavelength dispersive spectrometers (WDS) detector at the commonwealth scientific and industrial research organization (CSIRO), Clayton, Australia, was used to measure the chemical composition of all feldspar phases. Point Measurements were performed with an accelerating voltage of 15 kV, a probe current of 15 nA and a 5-10 nm-defocused probe size. Counting times were 40 s on peak and background. The elements were acquired using analyzing crystals PETH Ka for K, Ca, Ti, Cl, and TAP Ka for Na, Al, Si, and LDE1 for F Ka. The detection limits of Na, K, Al and Si in feldspar under such condition are below 185 ppm. Analytical errors for elemental concentrations are a function of the X-ray line intensities, background count rates, background positions, probe currents, counting times and the correction procedure applied. Repeated analyses of reference samples point to relative errors of 1 – 2 % for the major elements.

Nano-SIMS analysis

Nano-SIMS analysis was performed on polished epoxy mounts at the Centre for Microscopy,

Characterisation and Analysis (CMCA) at the University of Western Australia (UWA), using CAMECA Nano-SIMS 50L optimized as described by Wu et al.[1]. A Cs^+ ion source with spot size of approximately 50 nm was employed. Multiple electron multipliers record ion counts from the same sputtered sample volume at five masses simultaneously. Each region of interest was pre-sputtered using a beam current of 250 pA, to remove surface contaminants and implant Cs^+ ions into the sample matrix to achieve a steady state of secondary ion emission.

The electron gun was used for charge compensation because the samples were predominantly composed of insulating materials. Analysis areas ranged in size from 20×20 to 40×40 μm^2, the image sizes were 256×256 pixels. Nano-SIMS images were processed using ImageJ. The ion intensity of each trace element provides a simple, semi-quantitative expression of localized trace element enrichment.

Raman Spectroscopic Measurements

Raman mapping was conducted using a confocal WITec alpha 300 microspectrometer (WITec GmbH, Germany). Measurements were performed using 532 nm laser and 10 mW power with a ×50 objective. The scattered light was dispersed by a grating of 1800 grooves/mm on a CCD detector cooled to -60 °C. The WITec system was controlled using WITec Project 2.10 software. Several single points from mappings were chosen to show different phase spectrum characteristics. Frequency modes located between ~430-550 cm^{-1} correspond to O-Si-O and O-Al-O bending and stretching modes within the four-membered (Si_3AlO_{12}) tetrahedral rings.

Mineral liberation analyser (MLA)

Automatic whole sample mapping is performed by FEI QUANTA 650 FEG. Typical operating conditions use an accelerating voltage of 25 kV and spot size of 6-7. The typical magnification is around 250 times with the diameter of mapping area ranging from 30,000 mm to 8,000 mm, resulting in a total mapping time of around 3-5 hours. The analysis provided volume% of each phase; because of the similar density of sanidine, albite and K-feldspar, these volume% values were taken to also represent the weight % of these minerals. We made two sample blocks (>100 grains) from sample S3.4 (NaCl solution for 5 days) and conducted replicate measurements with MLA. Based on the replicate measurements, the calculated 1% standard error was used to represent the error of the results presented in Fig. 1 in the main text.

Supplementary Note: Thermodynamic modelling and GEMs Selektor Calculations

The chemical composition of the bulk fluid in equilibrium with sanidine at the respective experimental conditions was calculated using the GEMs Selektor software[2]. The MINES16 thermodynamic database (http://tdb.mines.edu/)[3], which contains standard thermodynamic properties for aqueous, gas and mineral species was chosen for our simulations. For aqueous electrolyte solutions, we use the extended Debye-Hückel (Helgeson) model to calculate activity coefficients of charged aqueous species[4]. For gas/fluid phase, the standard properties of pure real gaseous species are computed from the modified Peng-Robinson-Stryjek-Vera (PRSV)[5]. Details of all the models can be found in Wagner et al.[2]. Due to the asymmetric solvus system of alkali-feldspar, the multi-component Van Laar model[6] was used for the solid solution in calculations. Because at low temperatures there exists a miscibility gap between sodium and potassium feldspar end-members[2], two solid solution phases need to be used in the chemical system. For simplicity, two feldspar solid solution phases available in MINES16 were adopted but assuming that anorthite amount is negligible.

The staring composition of fluid and minerals used in Fig. 4 in the main text can be found in Supplementary Table 2. A titration model is used to model the reaction path of sanidine in

contact with pure water or Na-rich halide solution and calculate the evolution of the fluid and the minerals formed as a function of increase rock-fluid interaction by increasing the fraction of sanidine gradually to 1.

A conventional way to visualize Aq–SS equilibria and saturation states is based on plotting the Lippmann total solubility product[7]. In our case, 1 molar alkali feldspar solid solution with changing orthoclase to albite ratio to react with 40 g pure water, and the total solubility product can be written as:

$$\sum\prod = a(\mathrm{SiO_2})^3 * a(\mathrm{Al(OH)_4^-}) * (a(\mathrm{K^+}) + a(\mathrm{Na^+})) \qquad (1)$$

And the Lippmann diagram usually consists of two plots with a common ordinate $\log_{10}\sum\prod$ against two superimposed abscissas *y1* and *y2*, where *y1* is the aqueous ion activity fraction ($X(\mathrm{Na^+})$; solutus) and *y2* is the mole fraction in the solid (X(Ab); solidus). In the case of a miscibility gap, a segment of the solidus curve is metastable[8]. The boundary points between stable and metastable segments show the compositions of two solid solution phases in equilibrium with the peritectic composition of the aqueous phase[8].

Supplementary Table 1: Conditions of each experiments

Sample No.	S4-1	S4-3	S2-1	S3-4	S5-1	S2-2	S3-2
Reactants							
Sanidine (mmol)	0.11 ± 0.01						
Water (mmol)	2 ± 0.25						
Total halide (mmol)	0.15 ± 0.003						
Cl:(Cl+F) ratio	1	1	1	0	0	0	0.5
Solution	^{18}O-enriched	^{18}O-enriched	^{16}O-enriched	^{18}O-enriched	^{16}O-enriched	^{16}O-enriched	^{18}O-enriched
Reaction day(s)	1	3	5	1	3	5	5
Main Products (vol%)							
Sanidine	71.52 %	70.64 %	52.78 %	72.14 %	54.00 %	19.00 %	33.00 %
Albite	27.57 %	29.21 %	46.41 %	7.40 %	16.52 %	29.79 %	34.57 %
K-feldspar		0.08 %	0.41 %	20.05 %	26.88 %	51.01 %	31.15 %

Supplementary Table 2: Starting composition of each system used in modelling

	H_2O	NaCl solution	NaF solution	
Name	Quantity	Quantity	Quantity	units
Al_2O_3	5.64	5.64	5.64	g
CaO	1.00E-09	1.00E-09	1.00E-09	mol
K_2O	3.18	3.18	3.18	g
NaO	0.99	0.99	0.99	g
SiO_2	19.89	19.89	19.89	g
H_2O	40	40	40	g
NaCl	0	0.15	0	mol
NaF	0	0	0.15	mol
Cl	1.00E-09	0	1.00E-09	mol
F	1.00E-09	1.00E-09	0	mol

Thermodynamic modelling data source from: 1) Tagirov and Schott[9]; 2) Tagirov et al.[10]; 3) Miron al.[11]; 4) Schock et al.[12]; 5) Sverjensky et al.[13]; 6) Slop98.dat file

Supplementary Figure 1. Biotite only exist with albite in NaCl solution (S2-1) and fluorite and ilmenite coexist with albite in NaF solution (S2-2). Note the different accessory products formed in NaCl solution compared to NaF solution.

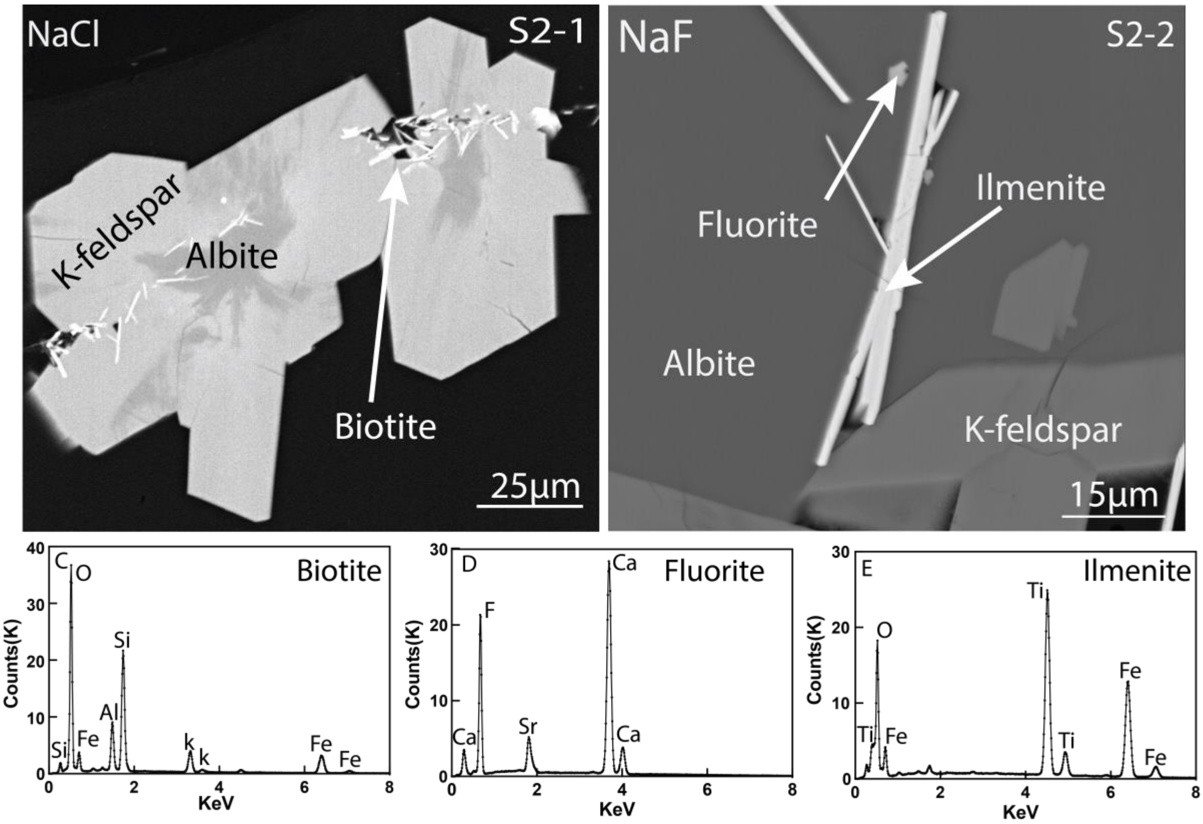

Supplementary Figure 2. Chemical compositions of three different feldspar phases, sanidine, albite and K-feldspar.

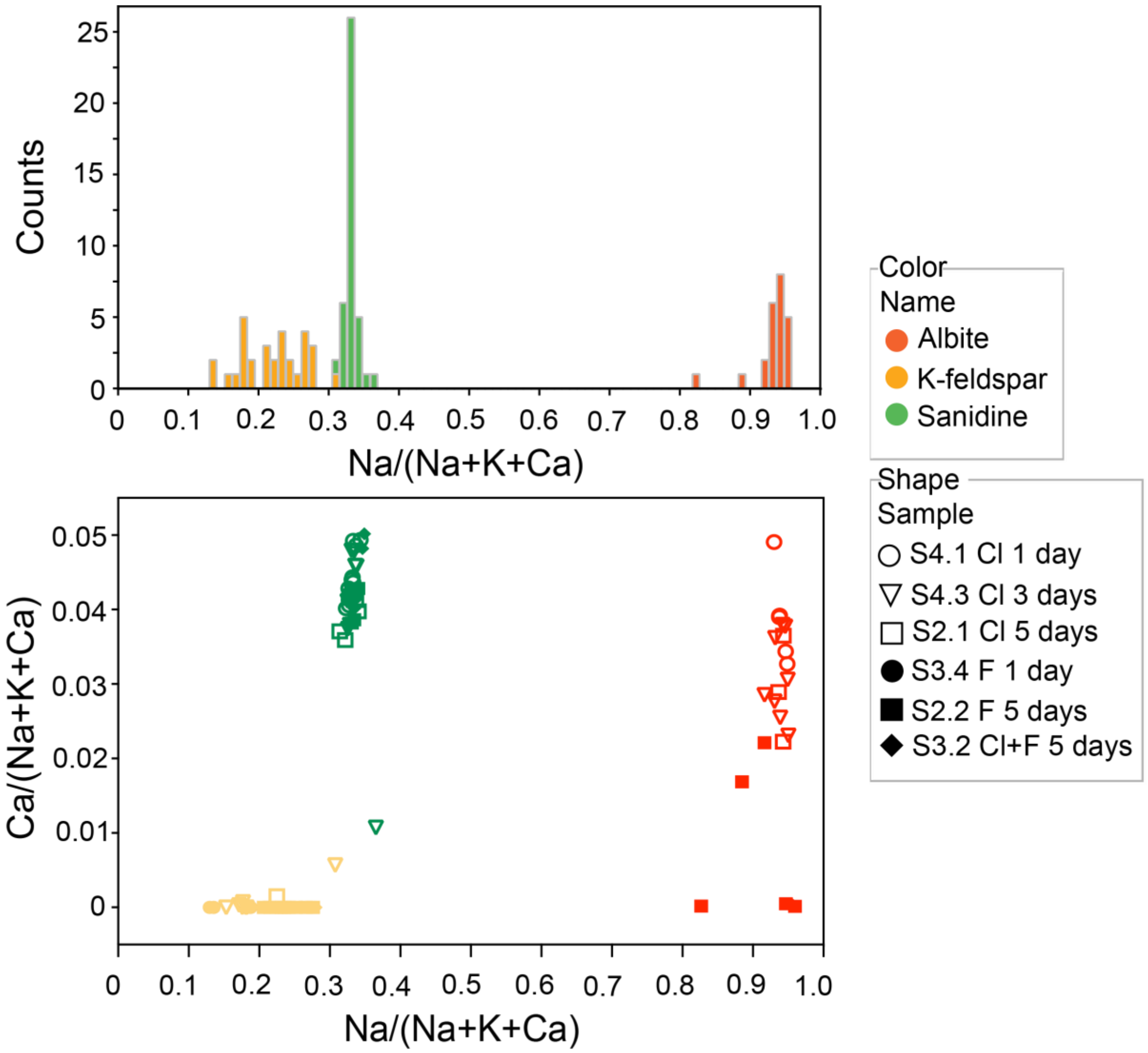

Supplementary Figure 3. Characteristic MLA mapping results highlight the varying feldspar fractions formed in NaCl solution (S2-1; Figs, A-B) and NaF solution (S2-2; Figs. C-D). A and C are the representative BSE images of reaction products. B and D are the corresponding MLA analysis. Blue, green and yellow represent sanidine, albite and K-feldspar, respectively. The small red colours observed in B and orange colours in D represent small biotite and fluoride grains, respectively.

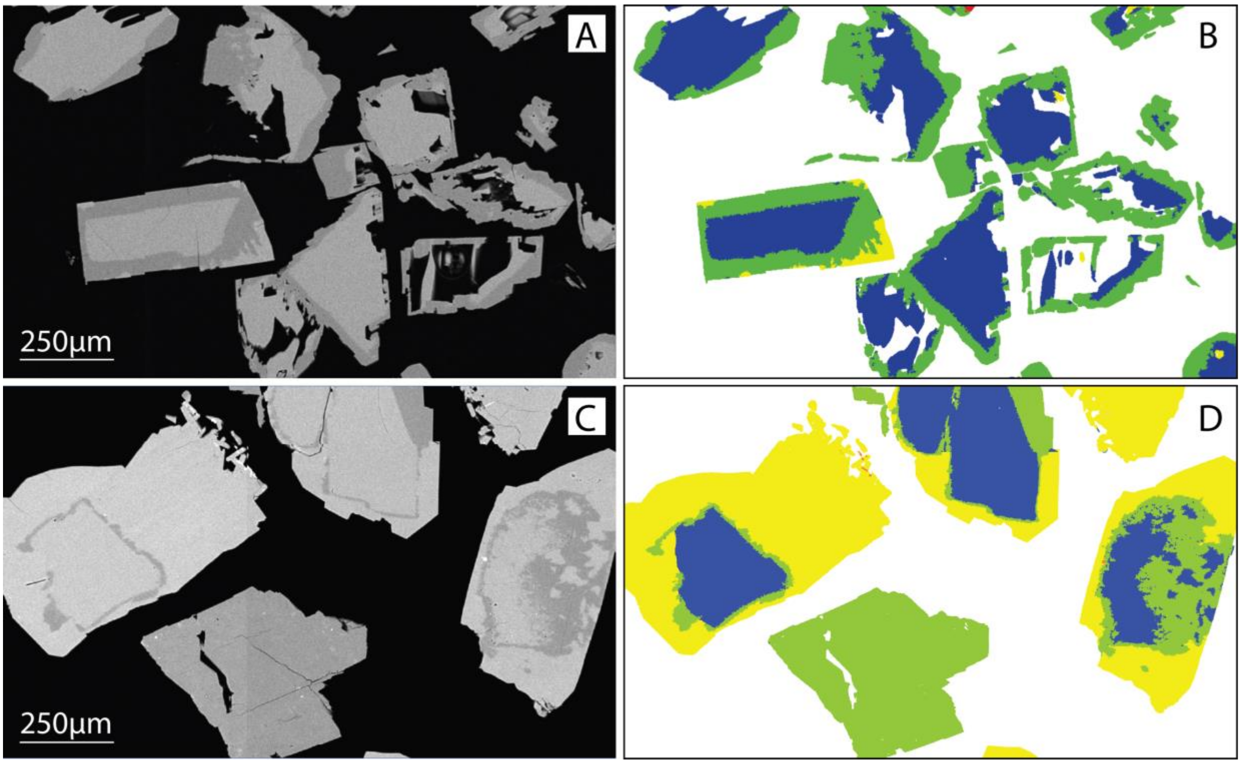

Supplementary Figure 4. Nano-SIMS analysis of line scan in reactions products formed in ^{18}O-enriched solutions with different reaction times. Note that the increasing trends of both ^{18}O and ^{16}O isotopes may be caused by charging or represents a diffusion effect.

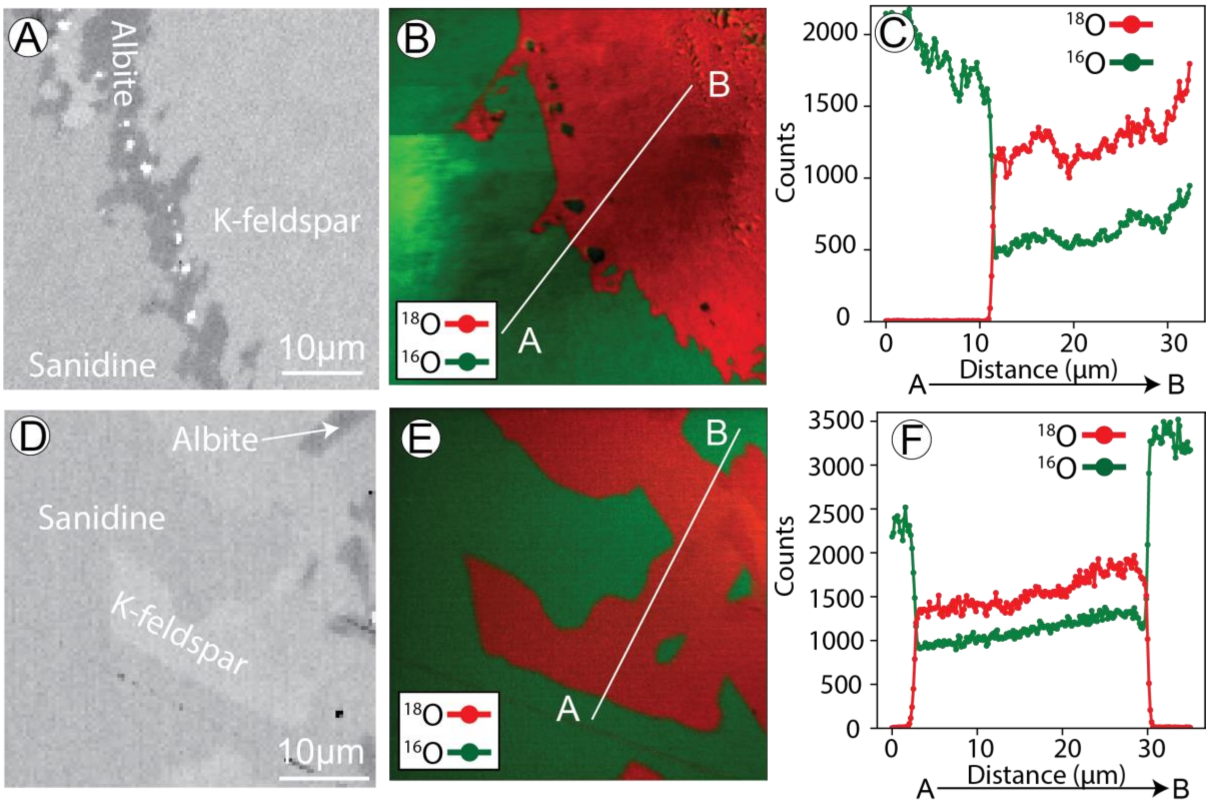

Supplementary Figure 5. Single point analysis of main Raman spectrum peaks of each phase. Solid lines represent albite and K-feldspar formed in ^{16}O-enriched solution, and dashed lines represent albite and K-feldspar formed in ^{18}O-enriched solution. Vertical lines represent the main peak near 515 cm^{-1} (compression of four-membered tetrahedral rings along c-plane) and 473 cm^{-1} (tetrahedral ring compression in a,b-plane) in each phase[14].

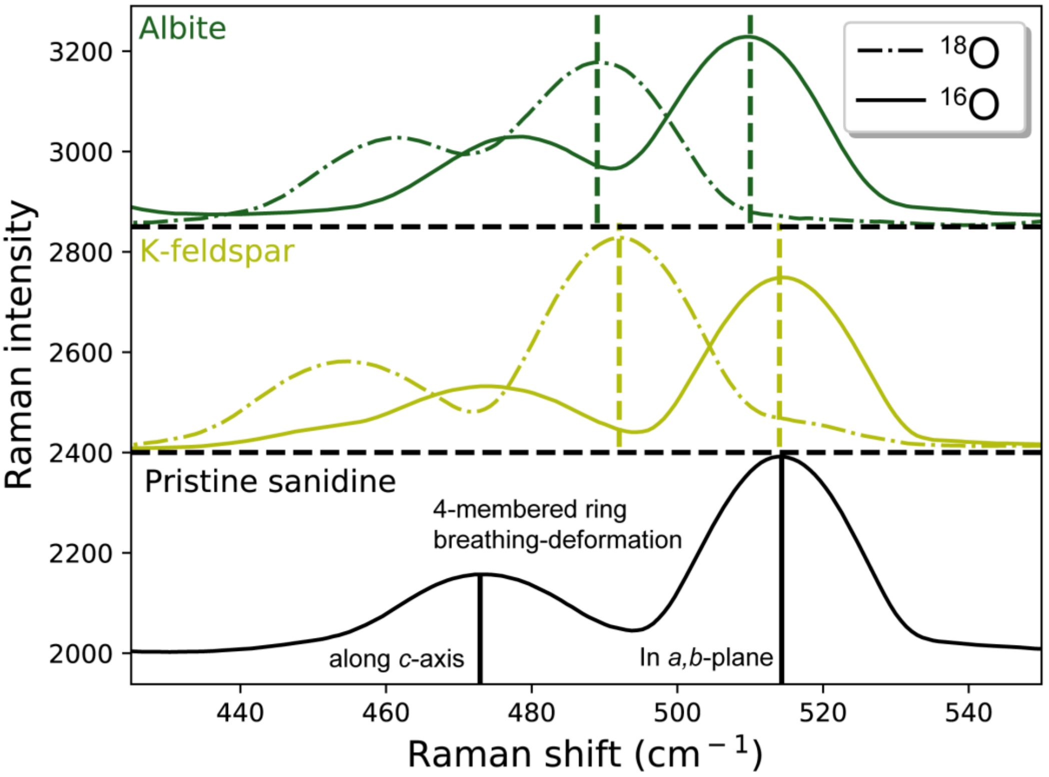

Supplementary Figure 6. Mappings of the position of the main Raman-active bending mode at 515 cm^{-1} in reaction products formed in in normal (Figs. A, B) and ^{18}O-enriched solution (Figs. C-F) with different reaction times (1 and 5 days). The blue and green colours of K-feldspar in D and F suggest the breakdown of the sanidine tetrahedral structural framework and incorporation of ^{18}O from water into albite and K-feldspar[15].

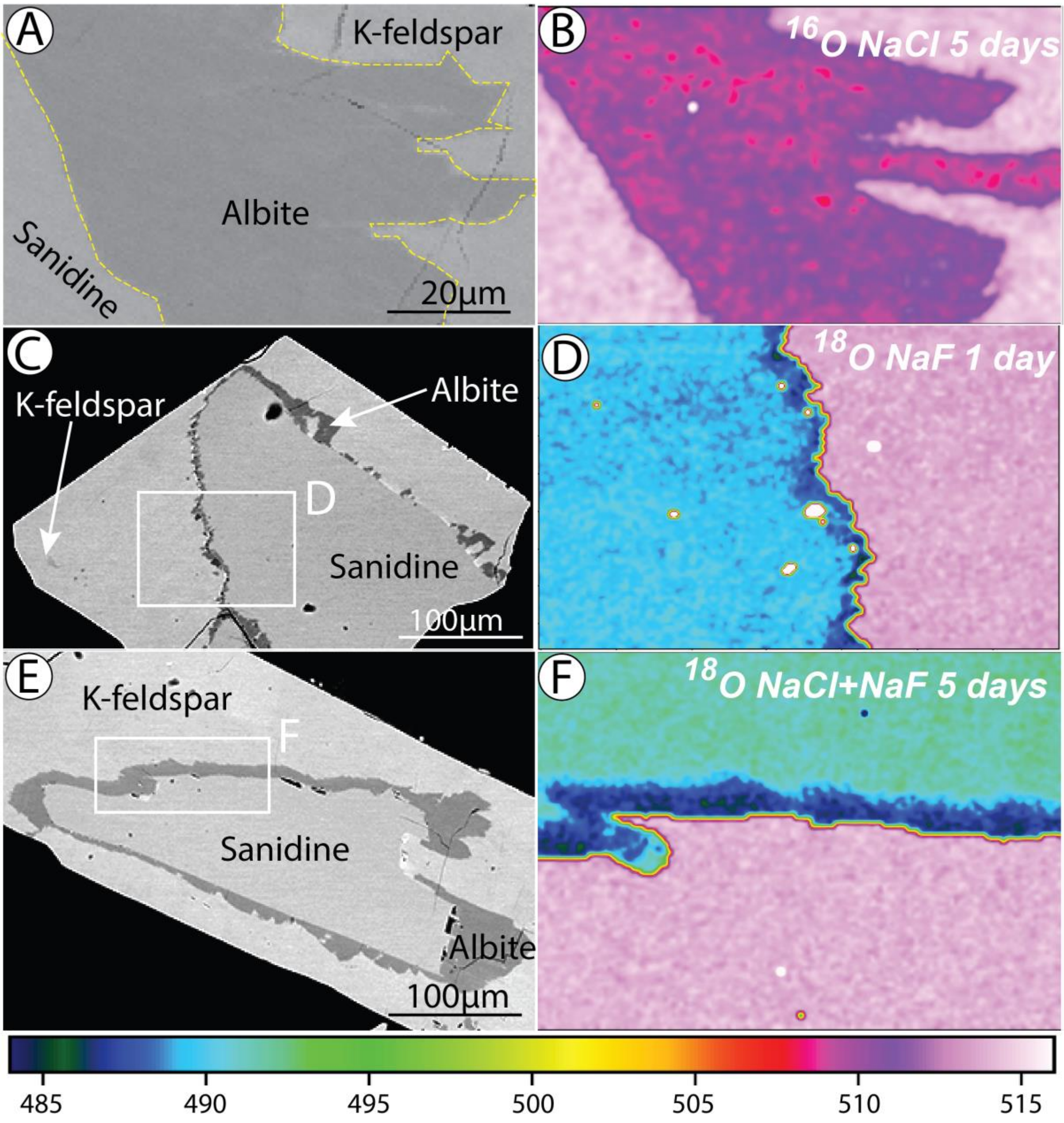

Supplementary References

1. Wu Y-F, Fougerouse D, Evans K, Reddy SM, Saxey DW, Guagliardo P, et al. Gold, arsenic, and copper zoning in pyrite: A record of fluid chemistry and growth kinetics. Geology 2019, 47(7): 641-644.
2. Wagner, T., Kulik, D. A., Hingerl, F. F., and Dmytrieva, S. V., 2012, GEM-Selektor geochemical modeling package: TSolMod library and data interface for multicomponent phase models: The Canadian Mineralogist, v. 50, no. 5, p. 1173-1195.
3. Gysi, A. P., 2017, Numerical simulations of CO_2 sequestration in basaltic rock formations: challenges for optimizing mineral-fluid reactions: Pure and Applied Chemistry, v. 89, no. 5, p. 581-596.
4. Helgeson, H. C., Kirkham, D. H., and Flowers, G. C., 1981, Theoretical prediction of the thermodynamic behavior of aqueous electrolytes by high pressures and temperatures; IV, Calculation of activity coefficients, osmotic coefficients, and apparent molal and standard and relative partial molal properties to 600 degrees C and 5kb: American journal of science, v. 281, no. 10, p. 1249-1516.
5. Stryjek, R. and Vera, J.H., 1986, PRSV—An improved peng-Robinson equation of state with new mixing rules for strongly nonideal mixtures: The Canadian Journal of Chemical Engineering, v. 64, no. 2, p. 334-340.
6. Holland, T. and Powell, R., 2003, Activity – composition relations for phases in petrological calculations: an asymmetric multicomponent formulation: Contributions to Mineralogy and Petrology, v. 145, no. 4, p. 492-501.
7. Glynn, P.D. and Reardon, E.J., 1990, Solid-solution aqueous-solution equilibria; thermodynamic theory and representation. American Journal of Science, v. 290, no. 2, p. 164-201.
8. Kulik DA, Vinograd VL, Paulsen N, Winkler B. (Ca, Sr) CO3 aqueous–solid solution systems: From atomistic simulations to thermodynamic modelling. *Physics and Chemistry of the Earth, Parts A/B/C* 2010, **35**(6-8)**:** 217-232.
9. Tagirov, B. and Schott, J., 2001, Aluminum speciation in crustal fluids revisited. Geochimica et Cosmochimica Acta, v. 65, no. 21, p. 3965-3992.
10. Tagirov, B.R., Zotov, A.V. and Akinfiev, N.N., 1997, Experimental study of dissociation of HCl from 350 to 500° C and from 500 to 2500 bars: Thermodynamic properties of HCl (aq): Geochimica et Cosmochimica Acta, v. 61, no. 20, p. 4267-4280.
11. Miron, G.D., 2016, Internally consistent thermodynamic database for fluid-rock interaction: tools, methods and optimization (Doctoral dissertation, ETH Zurich).
12. Shock, E.L., Sassani, D.C., Willis, M. and Sverjensky, D.A., 1997, Inorganic species in geologic fluids: correlations among standard molal thermodynamic properties of aqueous ions and hydroxide complexes: Geochimica et Cosmochimica Acta, v. 61, no. 5, p. 907-950.

13. Sverjensky, D.A., Shock, E.L. and Helgeson, H.C., 1997, Prediction of the thermodynamic properties of aqueous metal complexes to 1000 C and 5 kb: Geochimica et Cosmochimica Acta, v. 61, no. 7, p. 1359-1412.
14. McKeown, D.A., 2005, Raman spectroscopy and vibrational analyses of albite: From 25 °C through the melting temperature: American Mineralogist, v. 90, no. 10, p.1506-1517.
15. Niedermeier, D. R., Putnis, A., Geisler, T., Golla-Schindler, U., and Putnis, C. V., 2009, The mechanism of cation and oxygen isotope exchange in alkali feldspars under hydrothermal conditions: Contributions to Mineralogy and Petrology, v. 157, no. 1, p. 65.
