# Supplementary material for: Kinetically driven successive sodic and potassic alteration of feldspar
Source: Nat Commun. 2021 Jul 21;12:4435. doi: 10.1038/s41467-021-24628-1 (PMC8295371; doi:10.1038/s41467-021-24628-1)
Supplement: Supplementary file 3 — Description of Additional Supplementary Files [file 41467_2021_24628_MOESM3_ESM.docx]

**Description of Additional Supplementary Files**

File Name: Supplementary Data 1

Description: Electron microprobe analysis of sanidine, K-feldspar and albite
